# NAVIGATING THE PATH FROM PHD TO PI: INSIGHTS AND STRATEGIES FOR RESEARCH INDEPENDENCE

**DOI:** 10.1093/geroni/igae098.1523

**Published:** 2024-12-31

**Authors:** Marissa Schafer

**Affiliations:** Mayo Clinic, Rochester, Minnesota, United States

## Abstract

The path from PhD to PI can be a challenging uphill climb, and yet, there a are a multitude of steps one can take to increase the likelihood of securing an independent faculty research position. In this presentation, I will share insights from my path from PhD to PI. I will discuss key activities leading to research independence, including developing a research portfolio, engaging in skill-diversifying career development activities, and building supportive mentor-mentee relationships and networks. I will share my experience preparing for and participating in faculty job interviews, which include finding opportunities, giving job talks, and negotiating. As with research, there are many unknowns and many questions while looking forward to a career as an independent researcher. My goal is to share strategic opportunities and demystify processes to ultimately help others successfully navigate their path to research independence.

